# Supplementary material for: Subcellular Proteomics to Understand Promotive Effect of Plant-Derived Smoke Solution on Soybean Root
Source: Proteomes. 2021 Oct 2;9(4):39. doi: 10.3390/proteomes9040039 (PMC8544748; doi:10.3390/proteomes9040039)
Supplement: Supplementary file 1 [file proteomes-09-00039-s001.zip › new Supplemental Figures.pptx]

## Slide 1
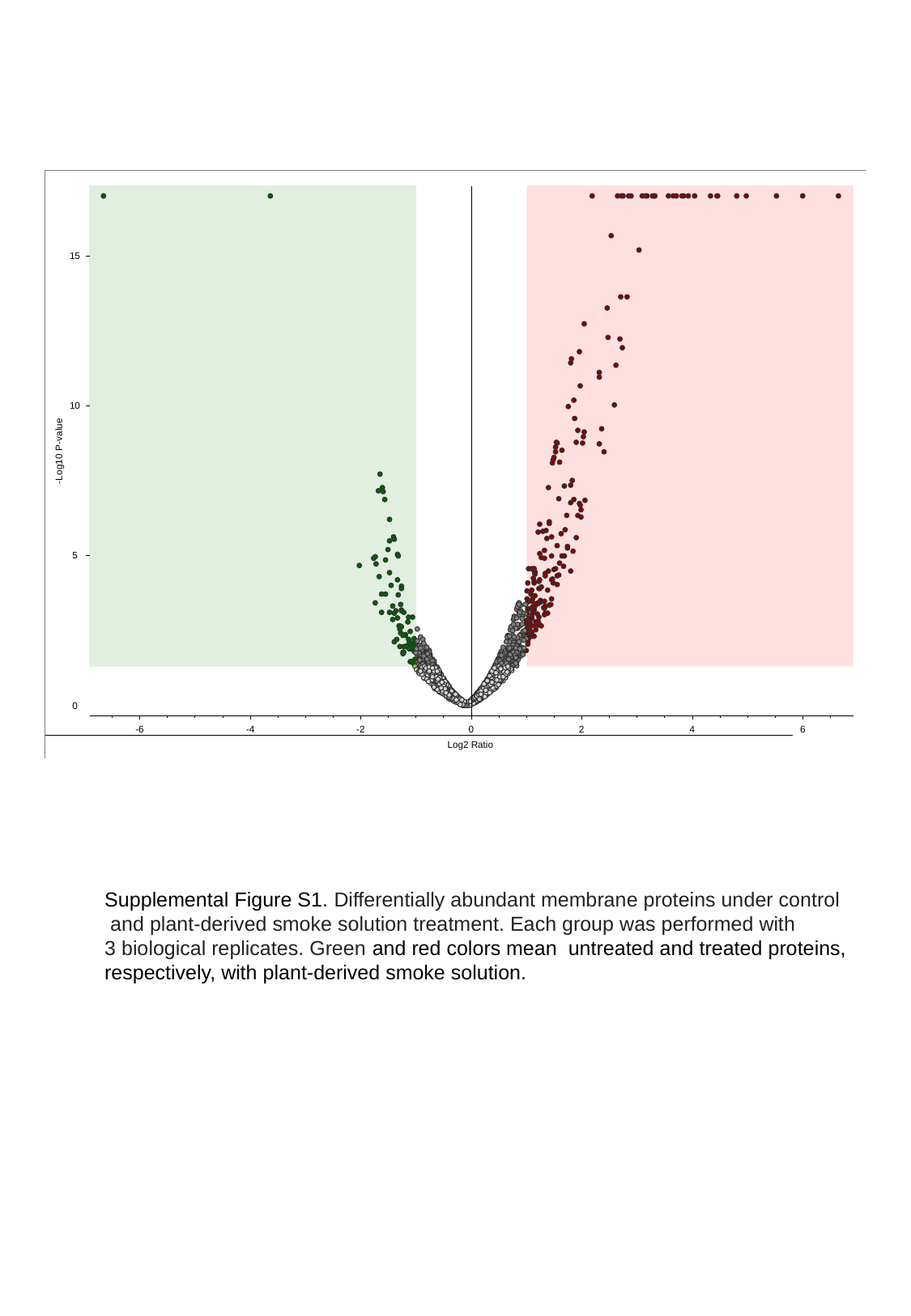

Supplemental Figure S1. Differentially abundant membrane proteins under control
 and plant-derived smoke solution treatment. Each group was performed with
3 biological replicates. Green and red colors mean untreated and treated proteins,
respectively, with plant-derived smoke solution.

## Slide 2
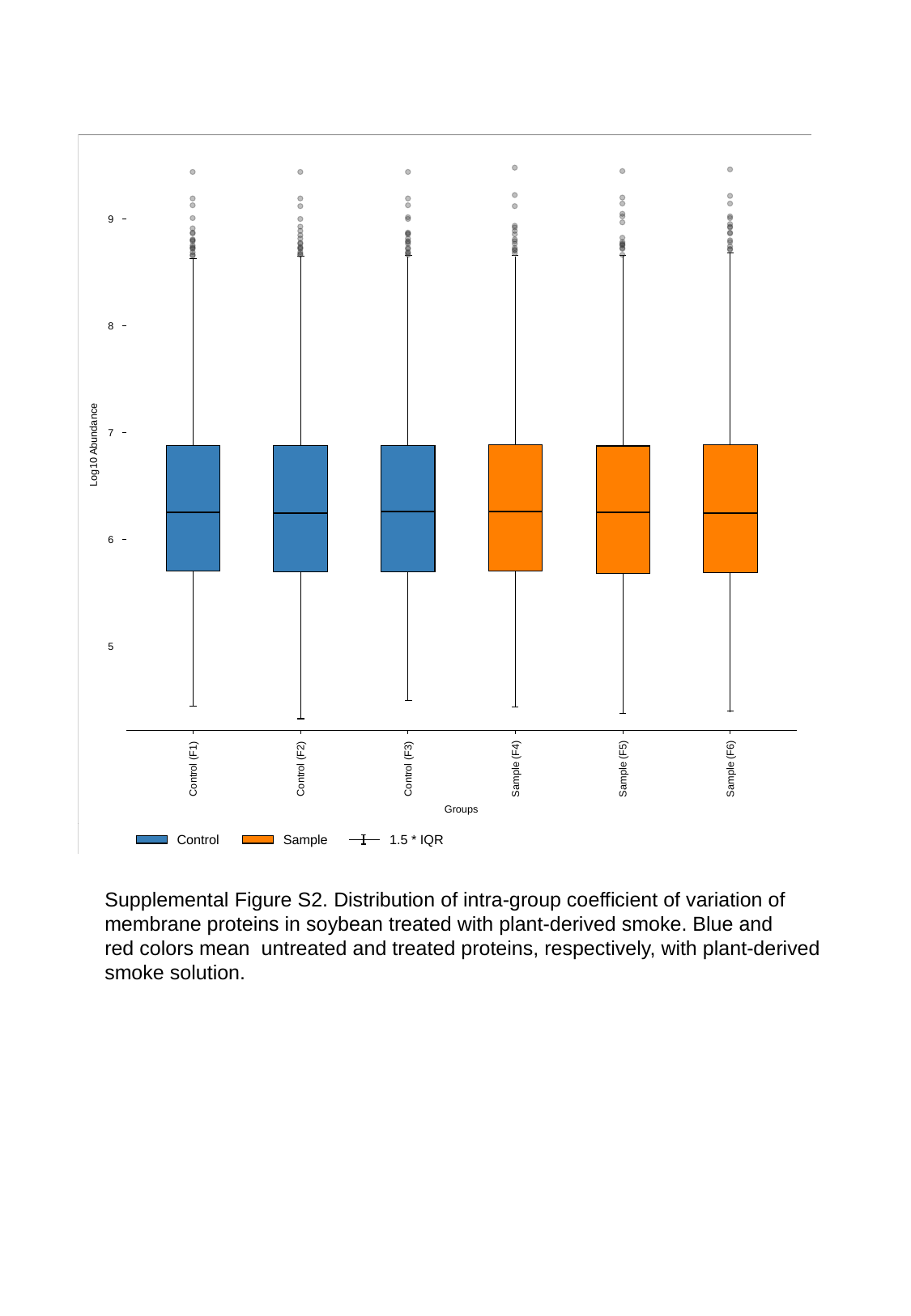

Supplemental Figure S2. Distribution of intra-group coefficient of variation of
membrane proteins in soybean treated with plant-derived smoke. Blue and
red colors mean untreated and treated proteins, respectively, with plant-derived
smoke solution.

## Slide 3
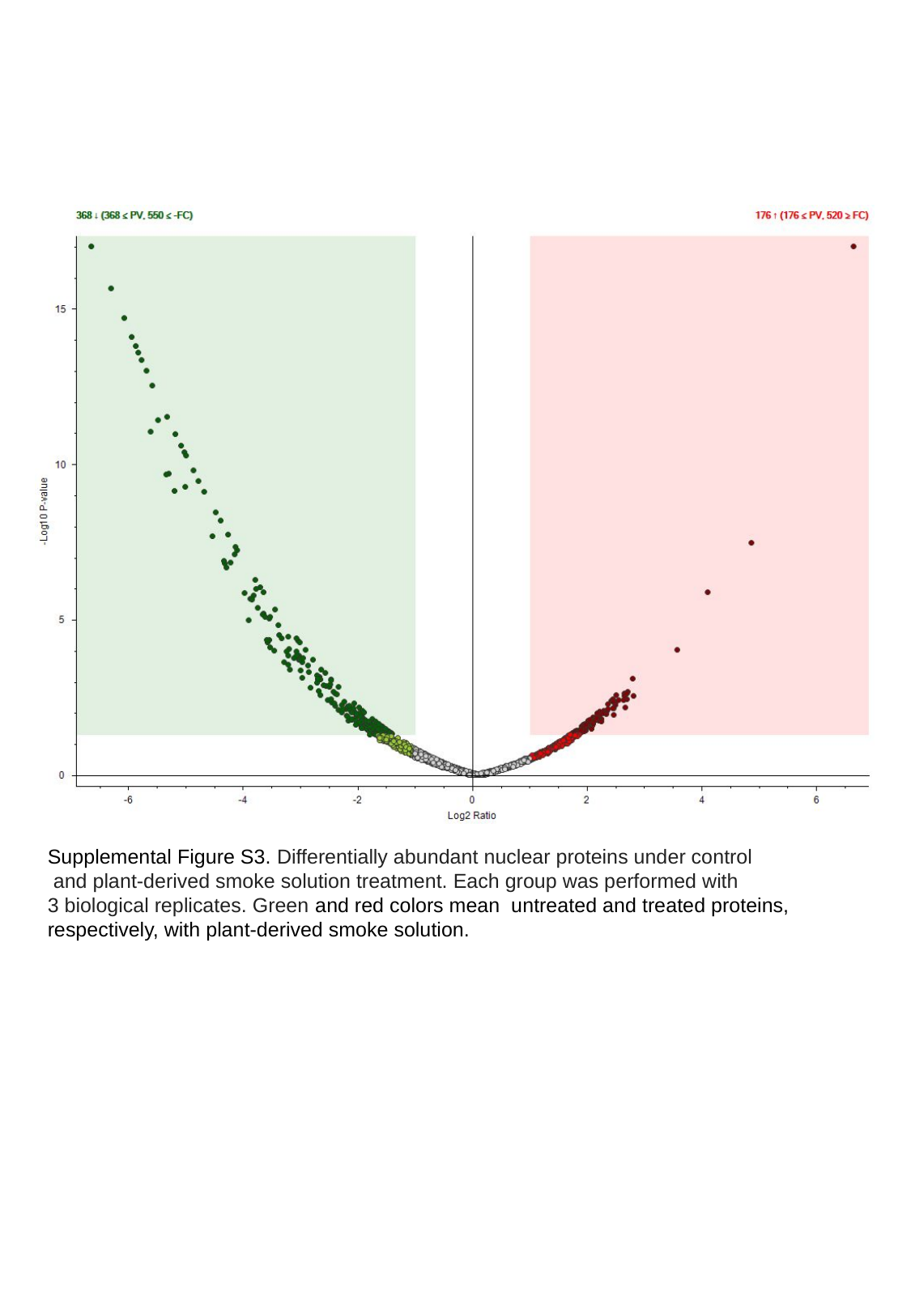

Supplemental Figure S3. Differentially abundant nuclear proteins under control
 and plant-derived smoke solution treatment. Each group was performed with
3 biological replicates. Green and red colors mean untreated and treated proteins,
respectively, with plant-derived smoke solution.

## Slide 4
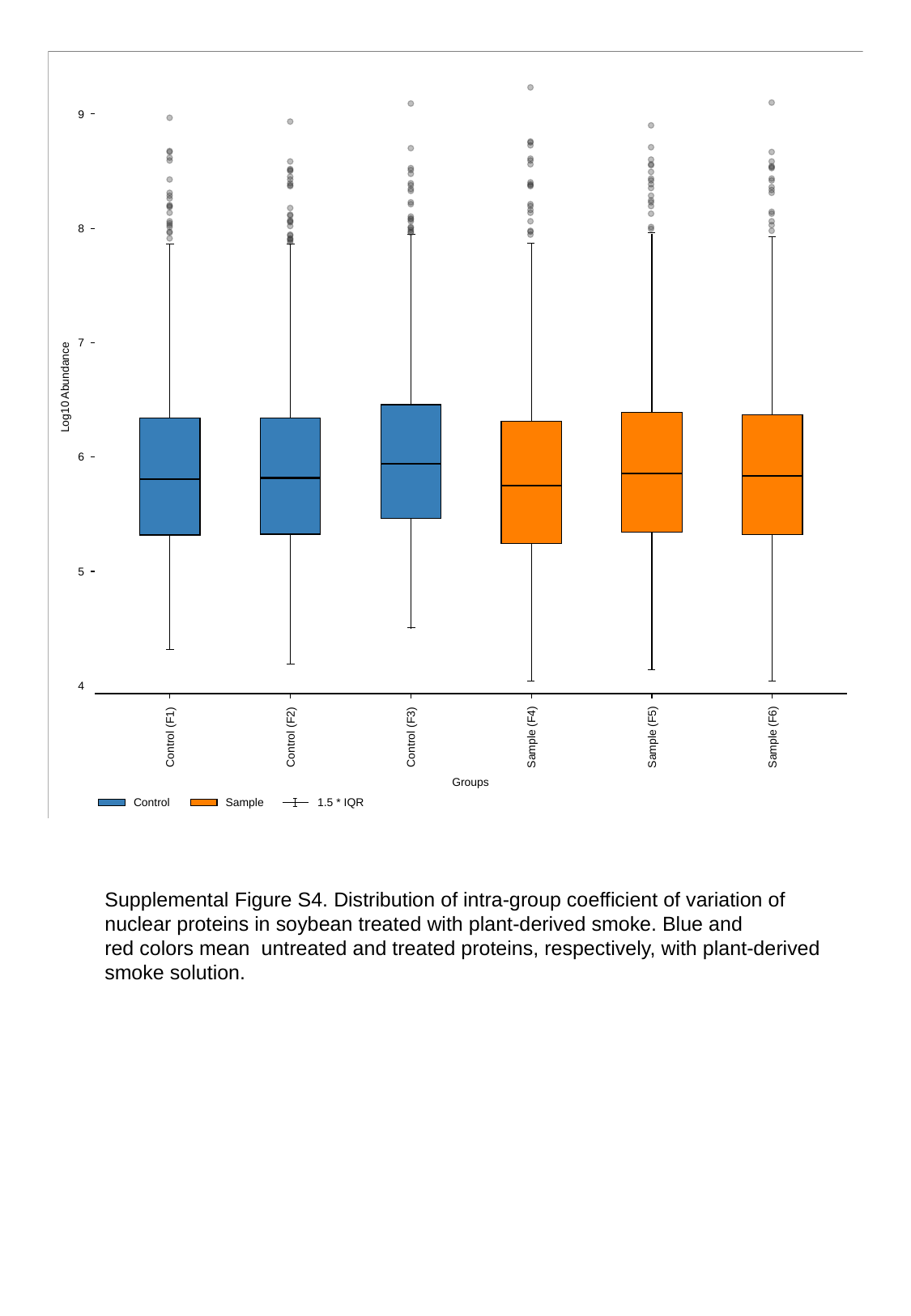

Supplemental Figure S4. Distribution of intra-group coefficient of variation of
nuclear proteins in soybean treated with plant-derived smoke. Blue and
red colors mean untreated and treated proteins, respectively, with plant-derived
smoke solution.

## Slide 5
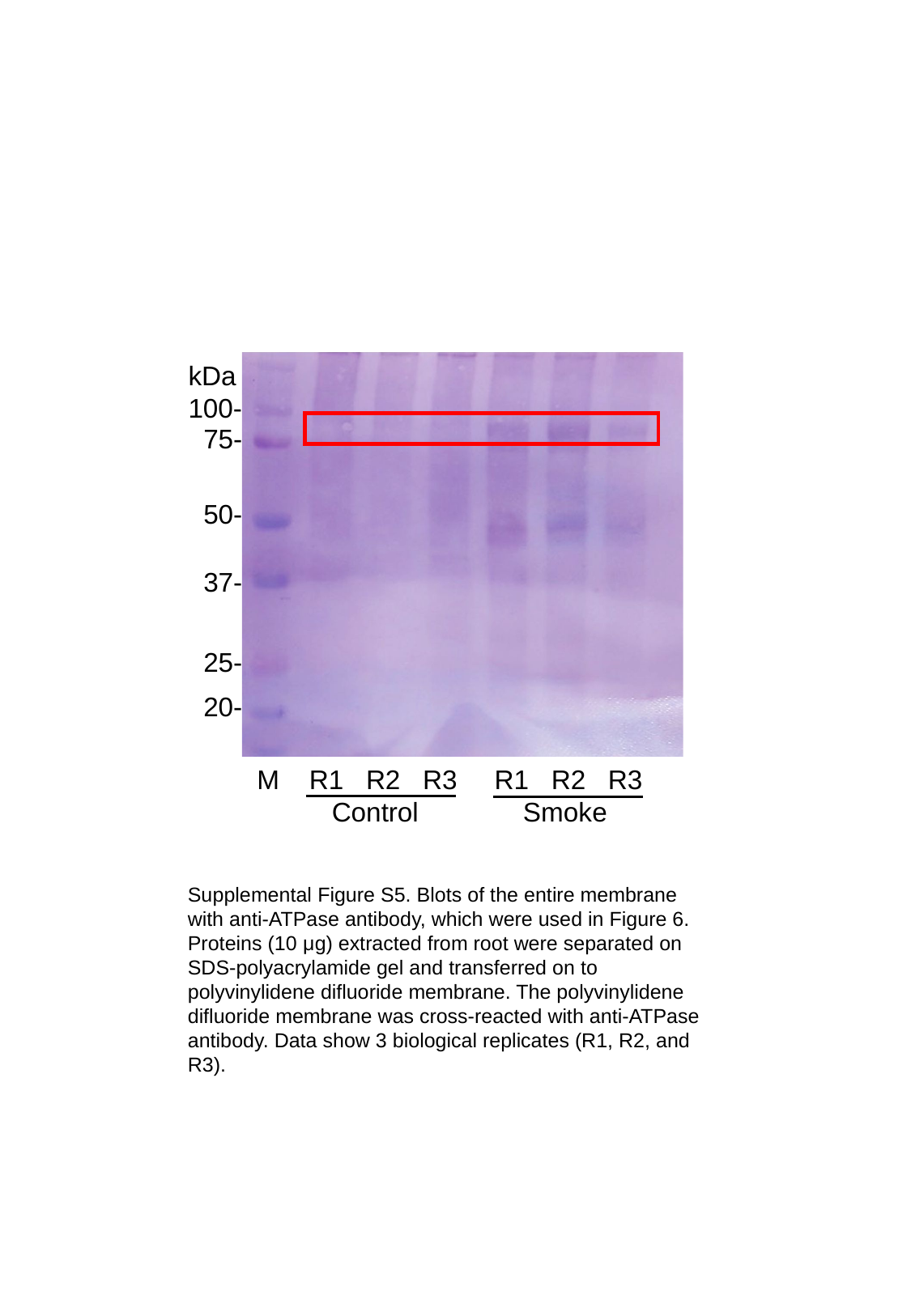

kDa
100-
75-
50-
37-
25-
20-
M R1 R2 R3 R1 R2 R3
 Control Smoke
Supplemental Figure S5. Blots of the entire membrane
with anti-ATPase antibody, which were used in Figure 6. Proteins (10 μg) extracted from root were separated on SDS-polyacrylamide gel and transferred on to polyvinylidene difluoride membrane. The polyvinylidene difluoride membrane was cross-reacted with anti-ATPase antibody. Data show 3 biological replicates (R1, R2, and R3).
