# Supplementary material for: Subcellular Proteomics to Understand Promotive Effect of Plant-Derived Smoke Solution on Soybean Root
Source: Proteomes. 2021 Oct 2;9(4):39. doi: 10.3390/proteomes9040039 (PMC8544748; doi:10.3390/proteomes9040039)
Supplement: Supplementary file 1 [file proteomes-09-00039-s001.zip › rev Supplemental Tables.pdf]

Supplemental Table S1. Materials and methods

Isolation of membrane fractions	A portion (2.0 g) of samples was chopped and ground in 1 mL of permeabilization buffer using a mortar and pestle. Homogenates were transferred to a tube, and incubated for 10 min at 4°C with constant mixing and centrifuged at 16,000 x g for 15 min at 4°C to pellet permeabilized cells. The supernatant containing cytosolic proteins was removed and transferred to a new tube. The pellet was resuspended in 0.5 mL of solubilization buffer. After pipetting, a homogeneous suspension was obtained and incubated for 30 min at 4°C with constant mixing. After centrifugation at 16,000 × g for 15 min at 4°C, supernatant containing solubilized membrane and membrane-associated proteins were sonicated with lysis buffer consisting of 7 M urea, 2 M thiourea, 5% CHAPS, and 2 mM tributylphosphine. After sonication, the homogenate was centrifuged at 12,000 x g for 30 min at 4°C, and the supernatant was collected as membrane proteins.
Isolation of nuclear fractions	A portion (2.0 g) of samples was ground with buffer. The homogenates were filtered through a double layer of filter mesh and centrifuged at 1,000 x g for 10 min at 4°C. The resulting pellet was resuspended in nuclei isolation buffer containing protease inhibitor mixture (Roche, Werk Penzberg, Germany) and layered on top of cushions containing 60% Percoll prepared in 1× nuclei isolation buffer and 2.3 M sucrose. After centrifugation at 3,200 x g for 30 min at 4°C, the middle layer was collected and washed with nuclei isolation buffer containing protease inhibitor mixture to remove Percoll and sucrose. The purified nucleus was vortexed with extraction buffer and sonicated. After sonication, the homogenate was centrifuged at 12,000 x g for 30 min at 4°C, and the supernatant was collected as nuclear proteins.
Immunoblot analysis	Quantified proteins (10 µg) were separated by electrophoresis on a 10% SDS-polyacrylamide gel and transferred onto a polyvinylidene difluoride membrane using a semidry transfer blotter (Nippon Eido, Tokyo, Japan). The blotted polyvinylidene difluoride membrane was blocked for 5 min in Bullet Blocking One regent (Nacalai Tesque, Kyoto, Japan). After blocking, the polyvinylidene difluoride membrane was cross-reacted with a 1: 1000 dilution of the primary antibodies for 1 h at room temperature. As primary antibodies, anti- H^{+}ATPase (Agrisera, Vannas, Sweden), anti-histone H3 (Abcam, Tokyo, Japan), anti-calnexin, and anti-ascorbate peroxidases antibodies were used. Anti-rabbit IgG conjugated with horseradish peroxidase (Bio-Rad, Hercules, CA, USA) was used as the secondary antibody. After 1 h incubation, signals were detected using TMB Membrane Peroxidase Substrate Kit (Seracare, Milford, MA, USA) following protocol from the manufacturer. The integrated densities of bands were calculated using Image J software (version 1.8; National Institutes of Health, Bethesda, MD, USA).
Protein enrichment, reduction, alkylation, and digestion	Methanol (400 µL) was added to each sample and mixed before addition of 100 µL of chloroform and 300 µL of water. After mixing and centrifugation at 20,000 x g for 10 min to achieve phase separation, the upper phase was discarded and 300 µL of methanol was added to the lower phase, and then centrifuged at 20,000 x g for 10 min (20). The pellet was collected and resuspended in 50 mM ammonium bicarbonate. It was reduced with 50 mM dithiothreitol for 30 min at 56°C and alkylated with 50 mM iodoacetamide for 30 min at 37°C. Alkylated proteins were digested with trypsin and lysyl endopeptidase (Wako, Osaka, Japan) at a 1:100 enzyme/protein ratio for 16 h at 37°C. Peptides were desalted with MonoSpin C18 Column (GL Sciences, Tokyo, Japan) and acidified with 1% trifluoroacetic acid.
Protein identification using nano-liquid chromatography mass spectrometry	The peptides were loaded onto the LC system (EASY-nLC 1200; Thermo Fisher Scientific) equipped with a trap column (Acclaim PepMap 100 C18 LC column, 3 µm, 75 µm ID x 20 mm; Thermo Fisher Scientific), equilibrated with 0.1% formic acid, and eluted with a linear acetonitrile gradient (0-35%) in 0.1% formic acid at a flow rate of 300 nL min^{-1}. The eluted peptides were loaded and separated on the column (NANO-HPLC capillary column C18, 3 µm, 75 µm × 150 mm; Nikkyo Technos) with a spray voltage of 2 kV (Ion Transfer Tube temperature: 275ºC). The peptide ions were detected using MS (Orbitrap Fusion ETD MS; Thermo Fisher Scientific) in the data-dependent acquisition mode with the installed Xcalibur software (version 4.0; Thermo Fisher Scientific). Full-scan mass spectra were acquired in the MS over 375-1,500 m/z with resolution of 70,000. The most intense precursor ions were selected for collision-induced fragmentation in the linear ion trap at normalized collision energy of 35%. Dynamic exclusion was employed within 15 sec to prevent repetitive selection of peptides.
Mass spectrometry data analysis	The MS/MS searches were carried out using SEQUEST HT search algorithms against the UniprotKB *Glycine max* (SwissProt TreEMBL, TaxID=3847, version 2017-10-25) using Proteome Discoverer (PD) 2.2 (version 2.2.0.388; Thermo Fisher Scientific). The workflow for both algorithms included spectrum files RC, spectrum selector, MASCOT, SEQUEST HT search nodes, percolator, ptmRS, and minor feature detector nodes. Oxidation of methionine was set as a variable modification and carbamidomethylation of cysteine was set as a fixed modification. Mass tolerances in MS and MS/MS were set at 10 ppm and 0.6 Da,

	respectively. Trypsin was specified as protease and a maximum of 2 missed cleavage was allowed. Target-decoy database searches used for calculation of false discovery rate and for peptide identification false discovery rate was set at 1%.
Differential analysis of proteins using mass spectrometry data	Label-free quantification was also performed with PD 2.2 using precursor ions quantifiler nodes. For differential analysis of the relative abundance of peptides and proteins between samples, the free software PERSEUS (version 1.6.14.0) (21) was used. Abundances of proteins and peptides abundances were transferred into log2 scale. Three biological replicates of each sample were grouped and a minimum of 3 valid values were required in at least one group. Normalization of the abundances was performed to subtract the median of each sample. Missing values were imputed based on a normal distribution (width = 0.3, down-shift = 1.8). Significance was assessed using Student's *t*-test analysis. Principal component analysis was performed with PD 2.2. The sequences of the differentially accumulated proteins were subjected to a BLAST query against the gene ontology database (http://www.geneontology.org/).
Measurement of ATP Contents	A portion (150 mg) of samples was homogenized in 100 μL of ADP assay buffer, centrifuged at 16,000 × g for 10 min at 4°C. For supernatant, sample deproteinization and neutralization were performed using Deproteinizing Sample Preparation Kit (Biovision). Extracts (50 μL) were added in 50 μL of reaction mixture containing ADP assay buffer, ADP probe, ADP converter, and ADP developer. After mixing, mixture was incubated at room temperature for 30 min in dark conditions and measured absorbance at 570 nm.

Supplemental Table 2. List of changed membrane proteins in soybean-root tip treated with plant-derived smoke solution compared with control

Accession no	Description	MP	MW [kDa]	calc. pI	Abundance Ratio	P-Value
A0A0R0JYY8	Tubulin beta chain	12	49.9	5.01	100	0.0000
A0A0R0KE60	Uncharacterized protein	7	30.1	4.31	100	0.0000
I1KC25	Acetolactate synthase	7	71	7.01	100	0.0000
I1JXH8	Uncharacterized protein	6	53.2	6.7	100	0.0000
I1J8S9	Uncharacterized protein	5	24.1	5.34	100	0.0000
I1L9X2	PlsC domain-containing protein	5	43.2	9.38	100	0.0000
I1KB56	Uncharacterized protein	5	37.7	9.58	100	0.0000
K7KTN4	Protein arginine N-methyltransferase	5	71.9	6.2	100	0.0000
I1N696	Uncharacterized protein	4	54.9	5.63	100	0.0000
I1N8B5	Uncharacterized protein	4	76	5.47	100	0.0000
A0A0R4J4S6	NADPH-protochlorophyllide oxidoreductase	4	43.2	8.78	100	0.0000
I1KPA0	Protein kinase domain-containing protein	4	54.8	5.43	100	0.0000
K7MBG3	Sucrose synthase	4	91.5	6.24	100	0.0000
K7MPG5	Uncharacterized protein	3	42.4	8.29	100	0.0000
I1JBN4	Uncharacterized protein	3	26	7.77	100	0.0000
I1L6A3	Uncharacterized protein	2	45.7	7.58	100	0.0000
Q949H4	Urease	2	90.7	6.18	100	0.0000
I1K4K8	Acyl-coenzyme A oxidase	2	75.6	8.9	100	0.0000
I1JMX7	Rubrerythrin domain-containing protein	2	49	8.22	100	0.0000
K7LLJ2	Uncharacterized protein	2	68.8	6.28	100	0.0000
I1JUP4	Oleosin	2	17.8	8.97	100	0.0000
P11828	Glycinin G3	2	54.2	5.97	100	0.0000
Q9XET1	Seed maturation protein PM31	2	17.7	6.55	100	0.0000
I1MCF0	Uncharacterized protein	2	45.2	6.43	100	0.0000
A0A0R0EDR0	Uncharacterized protein	2	15.7	7.56	100	0.0000
I1MT77	Uncharacterized protein	2	50.1	5.67	100	0.0000
I1KJJ6	Phytocyanin domain-containing protein	2	19.2	6.76	100	0.0000
O64458	34 kDa maturing seed protein	2	42.7	5.99	100	0.0000
P11827	Beta-conglycinin alpha' subunit	22	72.2	5.71	64.357	0.0000
A0A368UIC9	Uncharacterized protein	9	38.7	6.47	46.242	0.0000
I1N747	Oleosin	3	23.6	8.94	31.747	0.0000
A0A0R0HYM3	Uncharacterized protein	16	69.6	6.32	28.175	0.0000
P19594	2S albumin	5	18.4	5.34	22.006	0.0000
K7KTR9	Oleosin	2	22.6	8.7	21.778	0.0000
P29531	P24 oleosin isoform B	4	23.4	8.94	20.186	0.0000
I1L860	Uncharacterized protein	30	58	6.49	15.333	0.0000
Q9ZNZ4	Napin-type 2S albumin 1	5	17.8	6.38	14.458	0.0000
K7K4G2	Cupin_1 domain-containing protein	19	71.7	5.49	14.084	0.0000
I1LHP6	Cupin_1 domain-containing protein	15	81.7	5.34	13.108	0.0000
C6T586	Uncharacterized protein	3	22.7	5.58	12.672	0.0000
C6SZ13	Uncharacterized protein	3	15.8	9.36	11.951	0.0000
Q04672	Sucrose-binding protein	26	60.5	6.87	10.039	0.0000
I1M3M9	Uncharacterized protein	14	67.9	6.58	9.91	0.0000
A0A0R0JP17	Oleosin	3	21.4	10.27	9.791	0.0000
I1JF86	Uncharacterized protein	30	57.8	6.6	9.061	0.0000
I1L849	Uncharacterized protein	10	27.4	5.29	8.906	0.0000
Q42447	Maturation protein	8	25.6	6.52	8.576	0.0000
K7LGJ7	Aldo_ket_red domain-containing protein	4	36.5	7.49	8.224	0.0000
I1NGG4	Uncharacterized protein	9	26	5.02	7.488	0.0000
A0A0R0HVK7	Uncharacterized protein	5	22.2	6.52	7.229	0.0000

A0A0R0FSS4	Uncharacterized protein	4	23.8	6.27	7.063	0.0000
Q9XER5	Seed maturation protein PM22	7	16.7	5.36	6.761	0.0000
A0A0R0JWF4	Uncharacterized protein	3	84.8	7.42	6.646	0.0000
P01071	Trypsin inhibitor B	12	20	4.82	6.629	0.0000
C6T960	Uncharacterized protein	4	32	5.92	6.45	0.0000
K7LEQ5	Uncharacterized protein	9	26.6	6.8	6.305	0.0000
I1LPX6	Fructose-bisphosphate aldolase	6	42.8	7.33	6.163	0.0000
I1LE41	Uncharacterized protein	8	26.2	4.89	5.786	0.0000
I1JC17	Aldedh domain-containing protein	19	58.5	6.95	5.589	0.0000
C6T3A2	Bet v 1 domain-containing protein	5	16.7	4.93	5.533	0.0000
I1KAH2	Branched-chain alpha keto-acid dehydrogenase E1	6	55.3	6.99	5.315	0.0000
Q9ZTZ3	Reticulon-like protein	2	24.2	9.17	5.025	0.0000
Q43468	Hsp70-Hsp90 organizing protein 1	5	64.1	5.69	5.018	0.0000
A0A0R0L186	SHSP domain-containing protein	3	17.3	7.42	5.013	0.0000
I1M596	Lipoxygenase	37	97.2	6.67	4.565	0.0000
K7L4G7	Uncharacterized protein	4	95.3	6.48	4.183	0.0000
A0A0R0JD77	TENA_THI-4 domain-containing protein	3	33.6	7.87	4.154	0.0000
Q9ZTY1	35 kDa seed maturation protein	10	35.3	6.32	4.125	0.0000
I1N2Z5	Protein SLE1	5	12.2	5.38	4.098	0.0000
Q9SWB2	Seed maturation protein PM41	2	8.2	4.97	4.044	0.0000
I1L7P7	Cytochrome c oxidase subunit 5C	2	7.1	8.59	3.99	0.0000
A0A0R0IV26	Amidohydro-rel domain-containing protein	4	56.5	6.62	3.985	0.0000
K7KGC7	Sucrose synthase	15	92.2	6.13	3.937	0.0000
I1JFX0	Usp domain-containing protein	5	20.7	7.17	3.935	0.0000
C6TFC1	Non-specific lipid-transfer protein	2	12.4	8.63	3.911	0.0000
I1MQD2	BOWMAN_BIRK domain-containing protein	2	12.2	6.04	3.804	0.0000
Q42795	Beta-amylase	12	56	5.55	3.73	0.0000
K7LDT9	Uncharacterized protein	12	103.3	6.76	3.666	0.0000
I1JTU1	Branched-chain-amino-acid aminotransferase	7	42	7.11	3.648	0.0000
A0A0R0HA20	Uncharacterized protein	2	40	5.73	3.594	0.0002
C6TFE2	Uncharacterized protein	6	22.6	8.95	3.555	0.0000
B3TDK6	Lipoxygenase	49	96.7	6.58	3.515	0.0000
A0A0R0HAM0	Uncharacterized protein	4	41.8	6.52	3.498	0.0000
A0A0R0H569	Lipoxygenase	43	92.8	6.48	3.485	0.0000
I1M396	Knot1 domain-containing protein	2	8.5	7.84	3.485	0.0000
A0A0R0KSS1	PKS_ER domain-containing protein	3	37.9	6.27	3.479	0.0009
A0A0R0J965	Uncharacterized protein	9	34.4	6.9	3.381	0.0000
I1LNN8	DUF4149 domain-containing protein	2	39.9	9.29	3.354	0.0002
I1KVT5	Uncharacterized protein	2	15.5	6.01	3.35	0.0002
C6T0L2	Protein SLE3	4	11.1	6.8	3.326	0.0000
O23957	Dehydrin	3	17.3	9.23	3.237	0.0000
A0A0B5E8V1	Tau class glutathione S-transferase	4	25.9	6.7	3.147	0.0000
A0A0R0KL81	Uncharacterized protein	2	80.9	7.14	3.124	0.0003
C6T1V2	SHSP domain-containing protein	3	17.7	6.81	3.1	0.0001
C6T1Y2	Ribulose bisphosphate carboxylase small chain	2	20	8.66	3.045	0.0005
K7MQ84	Asparagine synthetase [glutamine-hydrolyzing]	9	65.2	6.73	3.015	0.0012
I1LN49	Uncharacterized protein	5	27	7.06	3.011	0.0000
Q9S7N8	Seed maturation protein PM21	2	10.1	5.01	2.945	0.0013
I1K554	Knot1 domain-containing protein	2	8.4	9.16	2.94	0.0000
A0A0R4J594	Peroxidase	4	34.8	7.9	2.939	0.0001
G3E7M9	Annexin	19	35.9	6.96	2.927	0.0000
Q9SWB6	Probable bifunctional TENA-E protein	10	26	5.35	2.905	0.0000
I1KWA0	Uncharacterized protein	7	65.2	4.83	2.905	0.0007

I1L957	Uncharacterized protein	19	48.8	6.42	2.894	0.0000
I1JLC8	Protein SLE2	5	11.5	5.6	2.822	0.0000
A0A0R0LK39	Uncharacterized protein	3	27.2	6.89	2.821	0.0008
Q38JD2	Temperature-induced lipocalin	2	21.4	7.24	2.819	0.0021
Q39801	51 kDa seed maturation protein	24	51	7.11	2.806	0.0000
Q39873	Lea protein	20	49.4	7.52	2.781	0.0000
I1M307	Bulb-type lectin domain-containing protein	3	48.8	6.64	2.767	0.0015
I1JYK1	SHSP domain-containing protein	2	26	7.96	2.763	0.0063
I1LS05	Uncharacterized protein	5	45.8	7.02	2.761	0.0016
A0A0R0FFL6	Uncharacterized protein	7	57.3	7.53	2.756	0.0003
P26987	Stress-induced protein SAM22	6	16.8	4.82	2.742	0.0001
I1LSS9	Flavodoxin-like domain-containing protein	3	21.8	5.57	2.71	0.0092
Q01417	18 kDa seed maturation protein	2	17.6	9.58	2.68	0.0000
P04776	Glycinin G1	16	55.7	6.23	2.665	0.0000
C6TIE2	AB hydrolase-1 domain-containing protein	2	35.3	5.3	2.65	0.0009
Q9XET0	Seed maturation protein PM30	4	15.1	8.9	2.635	0.0000
A0A0R0ENV4	Uncharacterized protein	8	23.5	5.3	2.595	0.0001
I1JL80	Ferritin	6	29.1	6.38	2.56	0.0000
I1K6M2	Uncharacterized protein	5	23.8	7.87	2.551	0.0011
I1MFL4	PAP fibrillin domain-containing protein	4	33.3	5.86	2.544	0.0156
C6SWV3	Uncharacterized protein	5	27.6	6.1	2.539	0.0012
I1LLR5	Uncharacterized protein	6	27.7	5.71	2.526	0.0002
A0A0R4J3N3	SHSP domain-containing protein	2	18.2	7.5	2.514	0.0075
Q39871	Late embryongenesis abundant protein	13	50.6	6.67	2.512	0.0004
I1N1Y7	Uncharacterized protein	2	8.5	6.95	2.507	0.0111
K7MZJ0	Sucrose synthase	21	92.2	6.38	2.462	0.0001
I1J7G5	Uncharacterized protein	6	18.6	5.02	2.413	0.0003
K7MID0	Uncharacterized protein	8	96.9	6.39	2.407	0.0026
A0A0R0HYB1	Uncharacterized protein	7	51.6	8.34	2.405	0.0365
I1KB92	Alpha-galactosidase	3	47.6	6.24	2.394	0.0070
C6TH01	Uncharacterized protein	4	29.3	7.08	2.384	0.0080
I1MFN0	Fumarylacetoacetase	6	45.8	6.28	2.378	0.0030
A0A0R0GZ36	Uncharacterized protein	5	41.3	5.99	2.376	0.0003
C6TFW4	Bet_v_1 domain-containing protein	6	16.7	5.05	2.37	0.0016
I1KRJ7	Thioredoxin domain-containing protein	13	24.4	6.92	2.366	0.0000
I1JHQ7	Uncharacterized protein	5	59.9	7.69	2.339	0.0030
A0A0R4J681	Uncharacterized protein	13	31.8	7.44	2.334	0.0018
I1JXA0	Uncharacterized protein	19	90.8	5.24	2.325	0.0086
A0A0R0I4F6	Uncharacterized protein	14	31.8	7.36	2.312	0.0001
I1KSA2	Uncharacterized protein	4	19.5	6.11	2.312	0.0086
I1JAU2	Uncharacterized protein	2	62	5.68	2.25	0.0286
I1KNX5	Aldehyde dehydrogenase	2	59.7	7.34	2.246	0.0375
I1K5Y5	Malic enzyme	13	70	7.46	2.239	0.0051
P93164	Gamma-glutamyl hydrolase	4	37.7	6.54	2.236	0.0100
I1KNG0	Uncharacterized protein	8	55.6	6.9	2.232	0.0011
A0A0R0I960	Uncharacterized protein	3	27	7.06	2.224	0.0009
I1LLM7	Asparagine synthetase [glutamine-hydrolyzing]	10	65.2	6.64	2.223	0.0010
I1JGR5	Uncharacterized protein	19	71.6	5.31	2.222	0.0087
I1JBQ4	DUF1338 domain-containing protein	7	41.6	7.93	2.218	0.0020
I1M988	Uncharacterized protein	4	8.7	9.32	2.217	0.0018
C6T0B7	Glycine cleavage system H protein	3	21.9	6.2	2.214	0.0104
I1JL98	Uncharacterized protein	5	18.5	5.38	2.203	0.0007
C6T374	VOC domain-containing protein	4	15.4	5.69	2.201	0.0009

I1LRD2	Stress-response A/B barrel domain-containing protein	5	12.8	6.52	2.195	0.0015
I1LC12	SHSP domain-containing protein	3	22.4	6.02	2.174	0.0155
I1KNL7	Uncharacterized protein	3	42.3	7.93	2.172	0.0064
I1NCA0	Uncharacterized protein	2	22.5	5.86	2.151	0.0187
K7MT91	Aldo ket red domain-containing protein	4	32.3	6.73	2.149	0.0114
I1J5A5	Aldedh domain-containing protein	22	58.4	6.84	2.146	0.0008
I1K268	Uncharacterized protein	2	40.9	7.36	2.141	0.0230
I1MT10	Uncharacterized protein	20	70.8	5.39	2.14	0.0074
B0M1A4	Catalase	18	56.7	7.25	2.139	0.0034
A0A0R4J3V5	MFS domain-containing protein	2	56.4	8.87	2.137	0.0365
Q9SWB4	Protein ADP-ribosyltransferase PARP3	21	91.6	5.59	2.126	0.0044
I1K3F6	FMN hydroxy acid dehydrogenase domain-containing protein	7	40.1	8.32	2.096	0.0320
C6TAT2	Tau class glutathione S-transferase	2	25.6	5.74	2.089	0.0250
I1MQD0	T-complex protein 1 subunit gamma	21	60.2	6.18	2.079	0.0289
K7KRB7	Amidase domain-containing protein	3	68.5	7.3	2.073	0.0460
I1J7H3	Ferritin	9	28.7	6.18	2.065	0.0007
I1KNN0	ATP-dependent 6-phosphofructokinase	4	51.1	6.71	2.063	0.0230
A0A0R0KKR3	Aldo ket red domain-containing protein	5	35.2	6.46	2.051	0.0409
I1LEP0	FAD-binding FR-type domain-containing protein	2	32	7.58	2.047	0.0125
I1M322	Uncharacterized protein	9	46.8	6.95	2.046	0.0125
C6SZX7	Glutathione peroxidase	5	18.5	7.09	2.043	0.0021
O81273	Biotin carboxylase	18	58.8	7.5	2.042	0.0276
I1LDP2	Uncharacterized protein	4	27.9	8.92	2.042	0.0077
I1K3K3	Urease	21	90.3	6.07	2.04	0.0114
O48560	Catalase-3	19	56.9	7.25	2.028	0.0446
I1JSY7	Plastocyanin	2	16.6	5.2	2.024	0.0433
A0A0R0FSB9	Uncharacterized protein	4	11.2	9.2	2.019	0.0165
I1M361	Uncharacterized protein	2	12.2	5.45	2.018	0.0035
K7LA74	Uncharacterized protein	2	11.4	9.66	2.017	0.0064
C6SVT0	Usp domain-containing protein	3	18	7.15	2.009	0.0319
K7LQ69	Poly [ADP-ribose] polymerase	18	91.1	5.43	2.007	0.0256
I1K467	Uncharacterized protein	18	59	6.51	1.987	0.0093
I1MJ34	Tau class glutathione S-transferase	9	25.5	6.61	1.981	0.0159
I1NFV9	Uncharacterized protein	3	28.5	6.16	1.965	0.0498
I1N786	Uncharacterized protein	10	43.5	6.87	1.914	0.0152
Q2I0H4	Glyceraldehyde-3-phosphate dehydrogenase	18	36.7	7.24	1.911	0.0193
I1MDY5	Amidohydro-rel domain-containing protein	9	57.2	6.25	1.895	0.0325
I1JIE1	Ferritin	10	27.7	5.96	1.874	0.0136
I1JF82	Uncharacterized protein	21	81.1	7.36	1.873	0.0096
A0A0R4J455	Uricase	14	35.1	8.29	1.857	0.0133
I1NBA5	Uncharacterized protein	5	26.4	9.17	1.854	0.0362
A0A0R0GUI6	Uncharacterized protein	5	19.9	8.88	1.84	0.0150
I1M0K3	Cysteine proteinase inhibitor	5	27.7	7.08	1.837	0.0358
I1LV54	Uncharacterized protein	9	46.6	6.8	1.836	0.0173
I1KSR6	Uncharacterized protein	12	39	5.22	1.826	0.0083
Q9FQD4	Glutathione S-transferase GST 24	6	24.8	6.07	1.822	0.0146
I1KRU6	FMN hydroxy acid dehydrogenase domain-containing protein	16	40.6	9.09	1.82	0.0092
P29756	Catalase-1/2	20	56.8	7.27	1.815	0.0093
I1M222	Uncharacterized protein	10	25.6	5.68	1.795	0.0104
A0A0R4J467	Uncharacterized protein	6	27.1	7.65	1.793	0.0413
I1J7A3	CBM20 domain-containing protein	3	51.6	4.42	1.787	0.0426
I1KZW8	PAP fibrillin domain-containing protein	5	33.6	5.31	1.785	0.0334
A0A0R0GFW6	Phosphoglycerate kinase	18	50	8.15	1.783	0.0381

A0A0R0JL19	Uncharacterized protein	15	41	6.4	1.781	0.0120
A0A0R4J3X4	SHSP domain-containing protein	5	25.6	7.49	1.767	0.0210
I1LVH0	PKS_ER domain-containing protein	4	40.2	7.46	1.752	0.0426
Q945U3	Acyl-coenzyme A oxidase	20	74.5	8.1	1.73	0.0193
C6T9Z5	Formate dehydrogenase, mitochondrial	21	42.8	6.77	1.719	0.0212
I1J9Q7	Glutamate dehydrogenase	16	44.8	6.4	1.708	0.0235
I1JET4	Uncharacterized protein	19	60.2	8.79	1.707	0.0332
I1JU43	NAD(P)-bd dom domain-containing protein	8	30.7	9.47	1.686	0.0495
A0A0R0KA84	Glyceraldehyde-3-phosphate dehydrogenase	18	36.8	7.58	1.654	0.0394
C6TCN5	Ferritin	10	28.1	6.04	1.633	0.0475
C6SYM8	Uncharacterized protein	3	21.9	5.12	1.633	0.0472
I1JF32	H15 domain-containing protein	2	27.6	10.62	0.48	0.0206
I1JKM1	Uncharacterized protein	3	32.3	5.17	0.457	0.0206
C6TI08	H15 domain-containing protein	2	19.8	10.92	0.454	0.0272
C6SY81	Uncharacterized protein	4	15.1	9.31	0.43	0.0149
I1JCX7	Uncharacterized protein	2	34.3	8.15	0.419	0.0026
I1MBW2	H15 domain-containing protein	4	18.9	10.26	0.418	0.0030
K7MRH9	Uncharacterized protein	15	68.7	7.81	0.417	0.0396
I1KHX1	Histone domain-containing protein	4	23.8	11.12	0.417	0.0125
I1N5V6	Uncharacterized protein	6	73.4	7.78	0.414	0.0095
K7MXM5	Uncharacterized protein	3	55	8.92	0.411	0.0461
A0A0R0KK01	Histone H2A	5	14.5	10.39	0.401	0.0003
I1LAX4	Uncharacterized protein	5	82.9	7.43	0.4	0.0048
A0A0R0HLG7	Histone domain-containing protein	5	30.5	10.1	0.399	0.0003
C6SYX1	Histone H2A	5	14.4	10.32	0.398	0.0016
A0A0R0FSB1	Uncharacterized protein	4	79.1	6.62	0.398	0.0208
C6SV65	Histone H2A	5	14.7	10.36	0.382	0.0001
A0A0R0F3E0	Histone H2A	4	15.5	10.71	0.379	0.0001
I1JE40	Uncharacterized protein	17	68.9	7.36	0.374	0.0102
C6SXI6	40S ribosomal protein S26	3	14.9	10.9	0.374	0.0243
C6TC09	Uncharacterized protein	3	31.5	9.54	0.368	0.0024
I1JHW6	H15 domain-containing protein	5	18.8	10.42	0.36	0.0001
C6SWA6	Histone H2A	4	15.7	10.67	0.359	0.0000
K7MFQ7	Uncharacterized protein	7	68.8	8.5	0.358	0.0148
A0A368UIC2	Protein kinase domain-containing protein	6	51.8	5.48	0.342	0.0046
I1JCJ8	Uncharacterized protein	3	31.4	10.08	0.342	0.0004
C6SZZ5	Histone H2A	4	14	10.05	0.34	0.0000
A0A0R0HEQ3	Histone H2B	4	16.2	10.08	0.334	0.0000
A0A0R0EX12	Histone H4	7	17.9	11.36	0.33	0.0000
I1LBM9	H15 domain-containing protein	2	19.9	10.86	0.324	0.0045
C6SZA4	Histone H3	5	15.3	11.3	0.319	0.0000
I1N9C6	Histone H2A	5	15.2	10.39	0.314	0.0000
C6SX28	Uncharacterized protein	2	14.2	9.79	0.302	0.0082
A0A0R0GXD9	Peroxidase	5	35.3	7.68	0.3	0.0003
C6T0Z6	Uncharacterized protein	5	16.6	9.99	0.295	0.0003
A0A368UHI2	Uncharacterized protein	17	57.7	5.33	0.01	0.0000
I1JU62	Transmembrane 9 superfamily member	13	73.1	7.62	0.01	0.0000
I1LKW4	Uncharacterized protein	12	62.4	9.11	0.01	0.0000
I1K5C0	TPR_REGION domain-containing protein	10	63.8	8.87	0.01	0.0000
I1JP84	Uncharacterized protein	6	170.8	7.03	0.01	0.0000
I1NGA5	Uncharacterized protein	5	37.8	9.25	0.01	0.0000
I1LFG4	TRANSKETOLASE_1 domain-containing protein	5	80.2	6.57	0.01	0.0000
I1KDE1	Nas2_N domain-containing protein	3	24.9	5.44	0.01	0.0000

I1JY96	Protein kinase domain-containing protein	3	113.6	7.11	0.01	0.0000
I1LYI2	Uncharacterized protein	3	17.4	9.7	0.01	0.0000
I1J8B3	Uncharacterized protein	2	18.9	5.39	0.01	0.0000
A0A0R0K3Q6	Peptidase A1 domain-containing protein	2	75.6	6.74	0.01	0.0000
I1KLY0	Uncharacterized protein	2	47.3	7.15	0.01	0.0000
K7KYN1	Uncharacterized protein	2	63.3	4.88	0.01	0.0000

Abbreviation means as follows: Cov, coverage; MP, matched protein; MW, molecular weight; Cal, calculated.

Supplemental Table 3. List of changed nuclear proteins in soybean-root tip treated with plant-derived smoke solution compared with control

Accession no	Description	MP	MW [kDa]	calc. pI	Abundance Ratio	P-Value
I1LPC3	Uncharacterized protein OS	5	134.7	6.58	100	0.0000
I1LYI2	Uncharacterized protein OS	3	17.4	9.7	100	0.0000
K7LGW1	Uncharacterized protein OS	3	60.4	9.2	100	0.0000
I1MD43	Peroxidase OS	3	37.7	8.4	100	0.0000
I1L3V3	Uncharacterized protein OS	3	48.9	5.71	100	0.0000
I1MKT0	Uncharacterized protein OS	2	54.1	8.44	100	0.0000
A0A0R0KBX5	Glutamine amidotransferase type-2 domain-containing protein	2	240.1	6.81	100	0.0000
C6TB35	Lipoyl-binding domain-containing protein	2	31.5	9.01	100	0.0000
I1JT76	Beta-galactosidase	2	82	8.13	100	0.0000
I1KMC5	Uncharacterized protein	2	31.5	5.94	100	0.0000
I1LYL9	Uncharacterized protein	2	67.6	8.41	100	0.0000
C6SWI0	Thioredoxin domain-containing protein	2	26.1	5.29	100	0.0000
A0A0R0E9J3	Uncharacterized protein	2	50.3	9.57	100	0.0000
A0A0R0KRF1	Uncharacterized protein	2	104.4	5.8	100	0.0000
I1LDU4	Uncharacterized protein	2	17.2	4.48	100	0.0000
A0A0R0FH68	NAD(P)-bd_dom domain-containing protein	2	43.8	8.48	100	0.0000
I1JDN7	Glyco_trans_2-like domain-containing protein	2	38.1	8.57	100	0.0000
I1M5Q4	ADP,ATP carrier protein	2	67.5	9.5	100	0.0000
C6TGY7	Proliferating cell nuclear antigen	3	29.5	4.79	29.341	0.0000
A0A0R4J2W1	Histone H2A	2	15.3	10.29	6.987	0.0082
C6T0A7	Uncharacterized protein	2	17.5	10.56	6.269	0.0272
I1KZ22	X8 domain-containing protein	3	51.3	9.31	6.221	0.0370
I1L5M2	Uncharacterized protein	3	42.8	8.6	5.876	0.0370
I1LZI3	Lysine--tRNA ligase	2	68.5	6.29	5.711	0.0260
C6TGI7	Uncharacterized protein	2	52.3	8.79	5.688	0.0397
I1LHP2	Uncharacterized protein	2	43.2	5.68	5.495	0.0342
I1JJS2	WD_REPEATS_REGION domain-containing protein	3	52.1	4.79	5.458	0.0411
C6TAF1	Uncharacterized protein	2	31.7	8.05	5.391	0.0377
I1JHW9	CBF domain-containing protein	10	64.8	8.27	5.143	0.0491
A0A0R4J4D6	Uncharacterized protein	7	71	5.21	0.241	0.0444
K7MIV6	Uncharacterized protein	5	23.6	7.33	0.213	0.0411
Q2I0H4	Glyceraldehyde-3-phosphate dehydrogenase	2	36.7	7.24	0.198	0.0146
A0A0R0I9M4	Annexin	8	35.5	8.76	0.194	0.0249
I1JSR9	MFS domain-containing protein	3	78.7	5.72	0.19	0.0227
I1MPM3	Uncharacterized protein	8	147.6	6.09	0.188	0.0201
G3E7M9	Annexin	16	35.9	6.96	0.182	0.0088
I1J728	Usp domain-containing protein	3	18.4	6.05	0.18	0.0122
I1LGG2	Glutathione peroxidase	3	18.5	6.19	0.178	0.0142
I1LP68	Bet_v_1 domain-containing protein	2	17.7	4.81	0.174	0.0359
I1LA46	Uncharacterized protein	2	30.6	6.68	0.169	0.0053
I1N5S0	Formate dehydrogenase, mitochondrial	2	42.8	6.77	0.167	0.0126
I1LV40	PKS_ER domain-containing protein	9	39.2	8.48	0.161	0.0041
B3TDK9	Lipoxygenase	4	96.3	6.81	0.16	0.0087
C6SVD0	Uncharacterized protein	2	12	5.31	0.158	0.0070
A0A0R0HCG7	PH domain-containing protein	12	484.4	6.16	0.155	0.0085
K7LQ69	Poly [ADP-ribose] polymerase	4	91.1	5.43	0.155	0.0083
I1J7H3	Ferritin	3	28.7	6.18	0.153	0.0065
I1JYA9	Superoxide dismutase	2	26.5	8.66	0.153	0.0106
I1KNN0	ATP-dependent 6-phosphofructokinase	14	51.1	6.71	0.146	0.0020
I1J4D5	Uncharacterized protein	3	26.6	5.9	0.142	0.0151

I1M138	Peptidase A1 domain-containing protein	4	51.2	8	0.138	0.0031
C6SVT0	Usp domain-containing protein	4	18	7.15	0.138	0.0052
Q39801	51 kDa seed maturation protein	11	51	7.11	0.134	0.0011
A0A0R0FFL6	Uncharacterized protein	2	57.3	7.53	0.13	0.0019
I1LRD2	Stress-response A/B barrel domain-containing protein	2	12.8	6.52	0.128	0.0024
Q9SWB4	Protein ADP-ribosyl transferase PARP3	3	91.6	5.59	0.126	0.0045
Q8RVH5	Basic 7S globulin 2	7	47.2	8.25	0.124	0.0021
I1M599	Lipoxygenase	6	92.7	7.01	0.124	0.0006
Q39873	Lea protein	9	49.4	7.52	0.123	0.0019
Q9FQD4	Glutathione S-transferase GST 24	5	24.8	6.07	0.123	0.0016
Q9XET0	Seed maturation protein PM30	3	15.1	8.9	0.123	0.0016
I1MT10	Uncharacterized protein	2	70.8	5.39	0.123	0.0021
I1M596	Lipoxygenase	21	97.2	6.67	0.122	0.0005
P0DO16	Beta-conglycinin alpha subunit 1	10	70.3	5.17	0.119	0.0004
C6T0B5	Uncharacterized protein	3	14	6.15	0.119	0.0012
A0A0R4J3X4	SHSP domain-containing protein	3	25.6	7.49	0.119	0.0015
A0A0R0FH00	Uncharacterized protein	3	71.2	5.22	0.116	0.0018
A0A0R0GZ36	Uncharacterized protein	4	41.3	5.99	0.109	0.0010
I1JIE1	Ferritin	3	27.7	5.96	0.108	0.0004
I1MNX4	Glutathione reductase	2	54	5.85	0.108	0.0029
I1LLR5	Uncharacterized protein	2	27.7	5.71	0.106	0.0011
K7LDT9	Uncharacterized protein	5	103.3	6.76	0.103	0.0025
A0A0R0FSB9	Uncharacterized protein	2	11.2	9.2	0.1	0.0004
P11828	Glycinin G3	9	54.2	5.97	0.097	0.0003
I1MJ34	Tau class glutathione S-transferase	4	25.5	6.61	0.096	0.0002
I1M1V8	Uncharacterized protein	11	63.4	6.11	0.093	0.0001
I1JL80	Ferritin	2	29.1	6.38	0.086	0.0001
C6T2N0	PRA1 family protein	2	20.4	9.52	0.086	0.0005
K7K3R6	Uncharacterized protein	2	36.8	6.39	0.085	0.0006
I1KNG0	Uncharacterized protein	3	55.6	6.9	0.082	0.0001
A0A0R4J467	Uncharacterized protein	4	27.1	7.65	0.08	0.0000
C6SYM8	Uncharacterized protein	3	21.9	5.12	0.08	0.0001
C6SWV4	Uncharacterized protein	5	18.8	6.32	0.077	0.0000
I1JL98	Uncharacterized protein	3	18.5	5.38	0.075	0.0000
K7KVC7	Knot1 domain-containing protein	2	8.6	8.46	0.074	0.0000
I1LDP2	Uncharacterized protein	7	27.9	8.92	0.073	0.0000
O23957	Dehydrin	2	17.3	9.23	0.071	0.0000
A0A0R4J3N3	SHSP domain-containing protein	3	18.2	7.5	0.07	0.0000
A0A0R0ENV4	Uncharacterized protein	4	23.5	5.3	0.068	0.0000
I1KMS6	Uncharacterized protein	5	19	7.5	0.064	0.0000
K7MZJ0	Sucrose synthase	9	92.2	6.38	0.058	0.0000
B3TDK6	Lipoxygenase	36	96.7	6.58	0.057	0.0000
I1JXY0	Uncharacterized protein	4	30.5	9.42	0.056	0.0000
I1M988	Uncharacterized protein	3	8.7	9.32	0.054	0.0000
A0A0R0I4F6	Uncharacterized protein	5	31.8	7.36	0.052	0.0000
I1L957	Uncharacterized protein	6	48.8	6.42	0.051	0.0000
K7LEQ5	Uncharacterized protein	5	26.6	6.8	0.05	0.0000
I1L0S5	Uncharacterized protein	3	19	6.93	0.05	0.0000
Q9SWB6	Probable bifunctional TENA-E protein	6	26	5.35	0.048	0.0000
C6T871	Uncharacterized protein	10	40.1	6.68	0.045	0.0000
I1KSR6	Uncharacterized protein	7	39	5.22	0.043	0.0000
A0A0R0H569	Lipoxygenase	25	92.8	6.48	0.039	0.0000
A0A0R0J965	Uncharacterized protein	7	34.4	6.9	0.037	0.0000

I1KRJ7	Thioredoxin domain-containing protein	10	24.4	6.92	0.034	0.0000
A0A0R0HVK7	Uncharacterized protein	8	22.2	6.52	0.031	0.0000
Q9ZNZ4	Napin-type 2S albumin 1	5	17.8	6.38	0.031	0.0000
C6T1V2	SHSP domain-containing protein	2	17.7	6.81	0.031	0.0000
K7K4G2	Cupin_1 domain-containing protein	13	71.7	5.49	0.03	0.0000
I1LHP6	Cupin_1 domain-containing protein	12	81.7	5.34	0.028	0.0000
A0A0R4J681	Uncharacterized protein	4	31.8	7.44	0.027	0.0000
Q9XER5	Seed maturation protein PM22	4	16.7	5.36	0.026	0.0000
I1JF86	Uncharacterized protein	18	57.8	6.6	0.025	0.0000
Q9ZTZ3	Reticulon-like protein	3	24.2	9.17	0.025	0.0000
I1JFX0	Usp domain-containing protein	7	20.7	7.17	0.022	0.0000
I1NGG4	Uncharacterized protein	7	26	5.02	0.021	0.0000
P11827	Beta-conglycinin alpha' subunit	23	72.2	5.71	0.019	0.0000
I1L860	Uncharacterized protein	21	58	6.49	0.018	0.0000
P01071	Trypsin inhibitor B	9	20	4.82	0.018	0.0000
P04776	Glycinin G1	13	55.7	6.23	0.017	0.0000
A0A0R0HYM3	Uncharacterized protein	20	69.6	6.32	0.016	0.0000
I1KBN9	Uncharacterized protein	4	29.5	9.66	0.015	0.0000
I1K554	Knot1 domain-containing protein	2	8.4	9.16	0.013	0.0000
I1M3M9	Uncharacterized protein	11	67.9	6.58	0.01	0.0000
I1L849	Uncharacterized protein	10	27.4	5.29	0.01	0.0000
I1LE41	Uncharacterized protein	7	26.2	4.89	0.01	0.0000
I1JRD0	Uncharacterized protein	6	110	5.62	0.01	0.0000
I1LBB9	Lipoxygenase	6	98.1	5.92	0.01	0.0000
A0A0B5E8V1	Tau class glutathione S-transferase	5	25.9	6.7	0.01	0.0000
P19594	2S albumin	5	18.4	5.34	0.01	0.0000
I1ND86	SHSP domain-containing protein	5	17.9	7.2	0.01	0.0000
I1LNN8	DUF4149 domain-containing protein	4	39.9	9.29	0.01	0.0000
Q9ZTY1	35 kDa seed maturation protein	4	35.3	6.32	0.01	0.0000
K7MBG3	Sucrose synthase	4	91.5	6.24	0.01	0.0000
I1MYB0	Uncharacterized protein	4	53.4	6.09	0.01	0.0000
C6SWV3	Uncharacterized protein	4	27.6	6.1	0.01	0.0000
C6TH01	Uncharacterized protein	4	29.3	7.08	0.01	0.0000
I1JXA0	Uncharacterized protein	3	90.8	5.24	0.01	0.0000
C6TFW8	Uncharacterized protein	3	25.9	7.3	0.01	0.0000
I1N8B5	Uncharacterized protein	3	76	5.47	0.01	0.0000
Q39872	PGmPM3	3	19.5	9.83	0.01	0.0000
A0A0R0I960	Uncharacterized protein	3	27	7.06	0.01	0.0000
P05478	18.5 kDa class I heat shock protein	3	18.5	6.09	0.01	0.0000
C6SYG6	Uncharacterized protein	2	21.8	4.84	0.01	0.0000
I1J5H1	Serine/threonine-protein phosphatase	2	34.9	5.01	0.01	0.0000
I1MCF0	Uncharacterized protein	2	45.2	6.43	0.01	0.0000
K7MID0	Uncharacterized protein	2	96.9	6.39	0.01	0.0000
Q42447	Maturation protein	2	25.6	6.52	0.01	0.0000
I1MDN4	Importin N-terminal domain-containing protein	2	118.7	5.02	0.01	0.0000
I1JUJ8	t-SNARE coiled-coil homology domain-containing protein	2	37.5	8.87	0.01	0.0000
I1KZW8	PAP fibrillin domain-containing protein	2	33.6	5.31	0.01	0.0000
I1MHY4	Uncharacterized protein	2	59.8	6.74	0.01	0.0000
P04405	Glycinin G2	2	54.4	5.58	0.01	0.0000
A0A0R0JWF4	Uncharacterized protein	2	84.8	7.42	0.01	0.0000
P02858	Glycinin G4	2	63.8	5.29	0.01	0.0000
K7LTG4	Uncharacterized protein	2	44.6	7.08	0.01	0.0000
I1JIE6	Uncharacterized protein	2	67.9	8.53	0.01	0.0000

I1JGB2	S-formylglutathione hydrolase	2	32.1	7.05	0.01	0.0000
K7MH28	Uncharacterized protein	2	58.3	6.49	0.01	0.0000
P25974	Beta-conglycinin beta subunit 1	2	50.4	6.24	0.01	0.0000
I1MAS2	Uncharacterized protein	2	43.5	6.24	0.01	0.0000
A0A0R0JP17	Oleosin	2	21.4	10.27	0.01	0.0000
M1FQK6	NADH-ubiquinone oxidoreductase chain 4	2	54.8	9.22	0.01	0.0000
I1JT73	Uncharacterized protein	2	49.1	7.56	0.01	0.0000
I1L6A3	Uncharacterized protein	2	45.7	7.58	0.01	0.0000
A0A0R0L186	SHSP domain-containing protein	2	17.3	7.42	0.01	0.0000
I1MQD2	BOWMAN_BIRK domain-containing protein	2	12.2	6.04	0.01	0.0000
I1KSL6	Uncharacterized protein	2	59.1	6.35	0.01	0.0000
C6TM03	Malate dehydrogenase	2	37.4	8.29	0.01	0.0000
I1K268	Uncharacterized protein	2	40.9	7.36	0.01	0.0000
K7MT91	Aldo_ket_red domain-containing protein	2	32.3	6.73	0.01	0.0000
I1LS05	Uncharacterized protein	2	45.8	7.02	0.01	0.0000
C6TP11	acidPPc domain-containing protein	2	36.3	7.47	0.01	0.0000
A0A0R0IVF1	Uncharacterized protein	2	48	8.22	0.01	0.0000

Abbreviation means as follows: Cov, coverage; MP, matched protein; MW, molecular weight; Cal, calculated.
